# Novel gastrointestinal disease in common marmosets characterised by duodenal dilation: a clinical and pathological study

**DOI:** 10.1038/s41598-020-60398-4

**Published:** 2020-03-02

**Authors:** Takayuki Mineshige, Takashi Inoue, Masahiko Yasuda, Terumi Yurimoto, Kenji Kawai, Erika Sasaki

**Affiliations:** 1 Department of Marmoset Biology and Medicine, Central Institute for Experimental Animals, Kawasaki, 210-0821 Japan; 2 Pathology Analysis Center, Central Institute for Experimental Animals, Kawasaki, 210-0821 Japan

**Keywords:** Experimental organisms, Experimental organisms, Microscopy, Microscopy

## Abstract

Common marmosets (*Callithrix jacchus*) are frequently used for biomedical research but gastrointestinal diseases have been major health problems to maintain captive marmosets. We have diagnosed a novel gastrointestinal disease in marmosets, as which we propose to call ‘marmoset duodenal dilation syndrome’; this disease is characterised by proximal duodenal obstruction and dilation. This study aimed to reveal the clinical and pathological findings of this syndrome and establish appropriate diagnostic imaging methods. Animals with the syndrome comprised 21.9% of the necropsy cases at the Central Institute for Experimental Animals in Kawasaki, Japan. The syndrome is characterised by clinical signs included vomiting, bloating, and weight loss. Grossly, all diseased animals exhibited significant dilation of the descending part of the duodenum, which contained a mixture of gas and fluid. The duodenal dilations were definitively diagnosed by contrast radiography. Moreover, a combination of plain radiography and ultrasonography was found to be a viable screening method for diagnosing duodenal dilation. The animals with duodenal dilation characteristically showed adhesions between the descending duodenum and ascending colon with chronic peritonitis. The cause of marmoset duodenal dilation syndrome remains unknown, but was likely multifactorial, including peritoneal adhesion, chronic ulcer, and feeding conditions in this study.

## Introduction

The common marmoset (marmoset) (*Callithrix jacchus*), is a New World primate that has been increasing number of used in biomedical and preclinical research in recent years^1–3^. Marmosets have several advantages as experimental animals including their small size (300–500 g), easy handling, and relatively rapid generational turnover^1–3^. Recent progress in transgenic and genome editing technology has also expanded the research use of marmosets^1,2,4^.

Gastrointestinal (GI) diseases are major problems to maintain captive marmosets^5–7^. Of these, the most important GI disease is Marmoset wasting syndrome (MWS), which is characterised by progressive weight loss, muscle atrophy, alopecia, diarrhoea, and enteritis^5,8,9^. Its prevalence has been reported to be as high as 28–60% in captive colonies around the world^5,9^. Several aetiologies have been suggested for MWS, including allergic, infectious, and autoimmune, but the exact pathogenesis remains unknown^9–12^. On the other hand, acute gastric dilation is well known as a GI disease in some nonhuman primates including marmosets and is characterised by voluminous gas and fluid retention in the stomach, vomiting, collapse, and death^13,14^.

Herein we describe a novel chronic GI disease with proximal duodenal obstruction and dilation in marmosets. This GI disease is characterised by repetitive vomiting, chronic bloating, and exhaustion, which result from the duodenal dilation and obstruction. This study aimed to (1) reveal the clinical and pathological findings of this novel GI disease, (2) establish a diagnostic imaging method using contrast radiology, and (3) determine a simple and easy screening test, without using contrast radiology. Finally, we discuss the possible pathogenesis and management of the syndrome.

## Results

### Retrospective analysis of necropsy records

Of the 224 marmosets necropsied at the Central Institute for Experimental Animals (CIEA; Kawasaki, Japan) during the 3 years between 2016 and 2018, 49 were diagnosed with duodenal dilation. Animals with duodenal dilation comprised 21.9% of the total non-experimental deaths necropsied during this period. These animals included 24 males and 25 females, and their ages ranged from 26 to 128 months (average: 76 months).

### Clinical and haematological findings

Detailed examinations were performed on marmosets that showed duodenal dilation and were necropsied between April 2017 and October 2018, and 14 animals were selected for clinical and pathological estimation (case nos. 1–14) (Table 1). We could not perform all imaging tests for the selected animals because of their clinical severity. Analyses were performed based on the availability of previously banked tissues, prior imaging tests, and/or stored serum samples; therefore, not all animals were included in every comparison. These animals included 7 males and 7 females, and their ages ranged from 32 to 101 months. Clinical signs of duodenal dilation included vomiting (11 of 14), weight loss (10 of 14), bloating (7 of 14), diarrhoea (6 of 14), and anorexia (2 of 14). Two marmosets showed severe vomiting after the administration of anaesthesia. All cases presented with a clinical history lasting over 1 month. Supportive treatments such as fluid infusion, nutritious food, and incubation were provided but were of limited value. Moreover, despite pharmacological treatment with famotidine, metoclopramide, and omeprazole, none of the diseased marmosets showed any significant improvement.

**Table 1:** Clinical and pathological findings for marmosets with duodenal dilation (Case Nos. 1–14) and control animals.

Case No.	Age at Death^a^	Sex	Body weight (g)	Clinical findings	Maximum diameter of the descending duodenum^b^ (mm)	Histopathological findings
1	40	F	336	vomiting, post-anesthesia vomiting, aspiration pneumonia, bloating, gastric dilation	15.6	NA
2	101	M	217	vomiting, weight loss	21.2	chronic peritonitis, ulcer in the inferior flexure, cholangitis/cholecystitis
3	98	F	257	diarrhoea, weight loss, emaciation, bloating, gastric dilation	28.0	chronic peritonitis, ulcer in the inferior flexure, cholangitis/cholecystitis
4	65	M	250	vomiting, weight loss, diarrhoea, bloating	13.3	NA
5	90	M	276	weight loss, acute collapse	16.0	NA
6	35	F	256	vomiting, weight loss, anorexia, bloating	13.8	NA
7	73	M	235	vomiting, weight loss	17.5	NA
8	85	M	258	weight loss, emaciation, anorexia, diarrhoea, bloating	20.0	chronic peritonitis, cholangitis/cholecystitis, chronic lymphocytic enteritis, pancreatic ductitis, liver abscess
9	47	F	308	vomiting, diarrhoea	19.5	NA
10	82	M	215	vomiting, weight loss, diarrhoea, gastric dilation	20.0	chronic peritonitis, ulcer in the inferior flexure, cholangitis/cholecystitis, chronic lymphocytic enteritis, pancreatic ductitis
11	65	F	313	vomiting, gastric dilation	23.8	chronic peritonitis, ulcer in the inferior flexure, cholangitis/cholecystitis
12	91	F	319	vomiting, diarrhoea, bloating	18.2	chronic peritonitis, ulcer in the inferior flexure, cholangitis/cholecystitis
13	53	M	267	vomiting, post-anesthesia vomiting, weight loss, emaciation, bloating, gastric dilation	27.5	chronic peritonitis, ulcer in the inferior flexure, chronic lymphocytic enteritis
14	32	F	250	vomiting, weight loss	12.8	chronic peritonitis, ulcer in the inferior flexure, chronic lymphocytic enteritis
Control animals^c^ (n = 22)	100 ± 32	M (n = 9), F (n = 13)	291 ± 56	—	5.8 ± 1.5	—

^a^Months, ^b^measured at necropsy, ^c^Mean ± standard deviation; Marmoset wasting syndrome (n = 8); *Clostridium difficile* infection (n = 2); tumour (n = 2); others (n = 10).

Blood tests revealed hypoalbuminaemia (<3.5 g/dl) in 8 of 12 animals, hypochloraemia (<95 mEq/l) in 4 of 12 animals, hypercreatininaemia (>0.5 mg/dl) in 4 of 12 animals, and high γ-glutamyltransferase (>10 IU/L) in 3 of 12 animals (Supplementary Table S1).

### Pathological findings

In normal marmosets, the duodenum is composed of 3 anatomical parts^3^: the descending duodenum (the long descending part located on the right side of the abdomen, connecting to the transverse part via the inferior flexure of the duodenum), the transverse duodenum (the short transverse part from right to left in the caudal half of the abdominal cavity), and the ascending duodenum (the short ascending part at the left side of the abdomen). The maximum diameters of the descending duodenum were 3.3–8.2 mm (average: 5.8 mm) in control animals.

**Figure 1:**
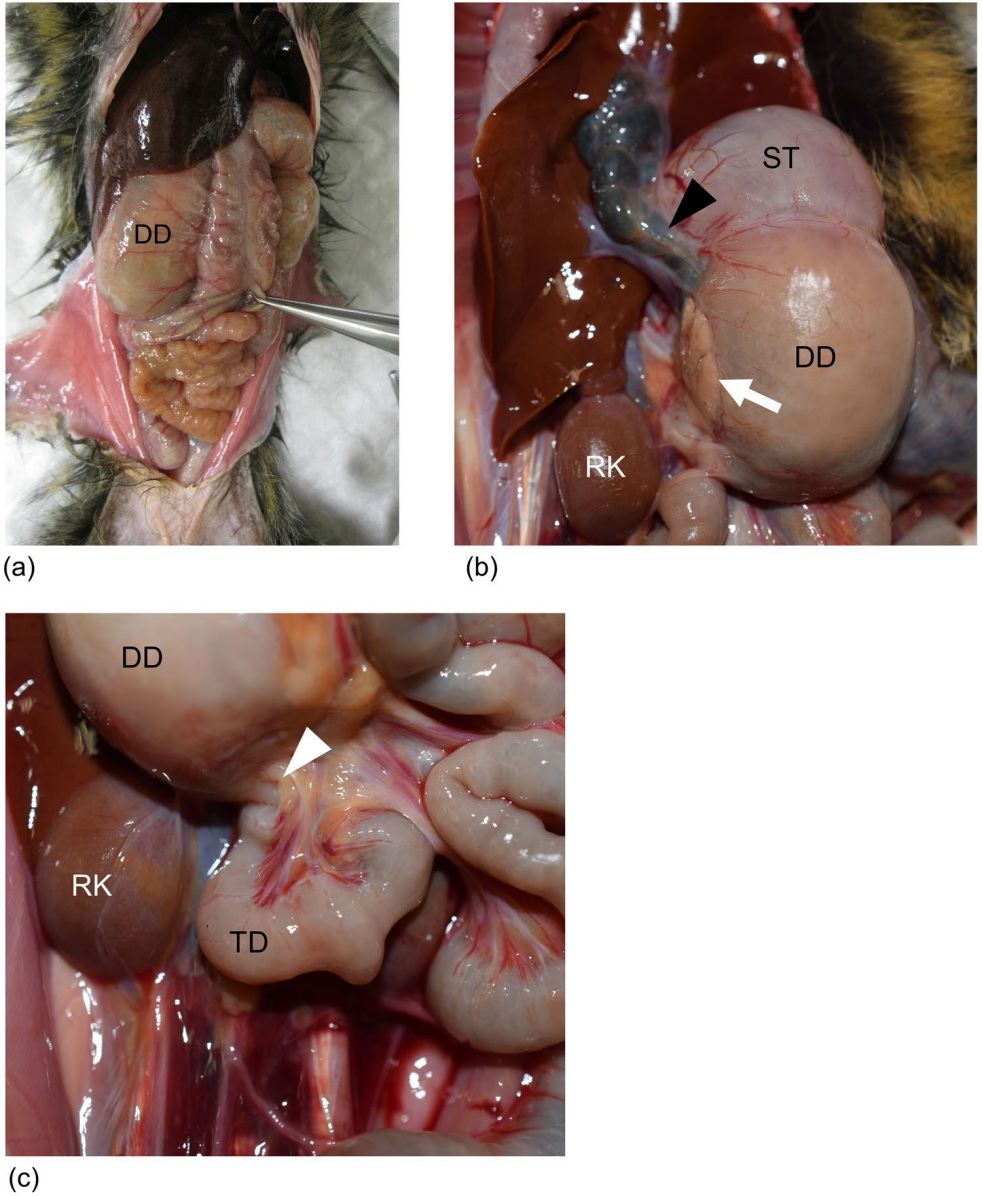
Macroscopic appearance of the dilated duodenum. (**a**) Diseased case (case no. 8). Marked dilation of the descending duodenum was seen. (**b**) Diseased case (case no. 10). Macroscopic appearance of the duodenum, common bile duct (arrowhead), and pancreas (arrow). The common bile duct entered the descending duodenum at one-third of its length. (**c**) Diseased case (case no. 10). Macroscopic appearance of the right side of the abdomen; abnormal flexion of the inferior flexure was seen (arrowhead). [DD: descending duodenum; TD: transverse duodenum; ST: stomach; RK: right kidney].

All 14 diseased animals exhibited significant dilation of the descending duodenum, which contained a mixture of gas and fluid (Fig. 1a,b). The maximum diameters of the descending duodenum were 12.8–27.5 mm (average: 19.1 mm). No dilation was evident in other parts of the duodenum or small intestine (i.e. the jejunum and ileum). In all diseased animals, there was no evident narrowing of the lumen in the inferior flexure of the duodenum; however, abnormal flexion of this region appeared to cause incomplete bowel obstruction (Fig. 1c). Duodenal compression by other organs (i.e. the superior mesenteric artery) was not observed at the inferior flexure. Gastric dilation was also observed in 5 of 14 animals. Pyogenic cholangitis was identified in 2 diseased marmosets (case nos. 3, 8), and *Escherichia coli* was isolated from the suppurative bile (case no. 8). Aspiration pneumonitis was a complication in 1 diseased case (case no. 1).

**Figure 2:**
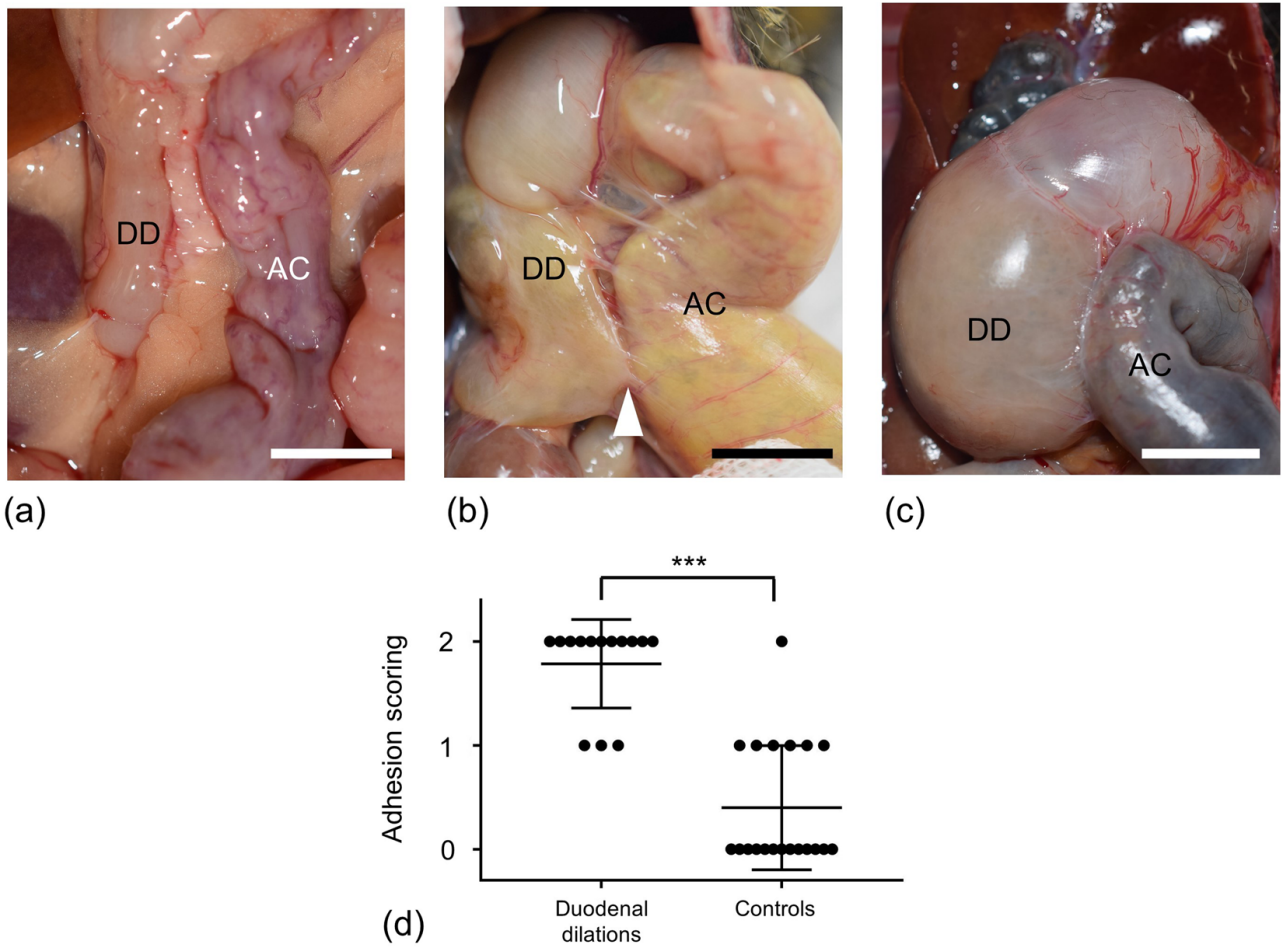
Macroscopic appearance of the duodenum and colon. (**a**) Control case. No adhesions were seen between the descending duodenum and ascending colon (adhesion score 0). (**b**) Control case. Partial adhesions were seen between the duodenum and colon (arrowhead) (adhesion score 1). (**c**) Diseased case (case no. 10). Tight and direct adhesions present between the duodenum and colon (adhesion score 2). [DD: descending duodenum; AC: ascending colon; Bar = 10 mm] (**d**) Animals with duodenal dilation showed significantly higher adhesion scoring than controls. The long horizontal line represents the mean and the short horizontal line represents the standard deviation (SD). ***P < 0.001. Unpaired t-test.

Disease cases characteristically showed marked adhesions between the descending duodenum and the ascending colon. In all marmosets including the controls, the ascending colon and descending duodenum were adjoined (Fig. 2a). In control animals, no or partial adhesions were seen between the descending duodenum and ascending colon (Fig. 2b), although one control case showed tight and direct adhesion without duodenal dilation. Diseased cases showed significantly higher adhesion scores than control animals (Fig. 2c,d). During necropsy inspection, detachment of the adhesion released the duodenal obstruction in 6 diseased cases (Nos. 1, 4–7, and 9). In the 8 remaining cases, the adhesions were preserved for histopathological analysis.

**Figure 3:**
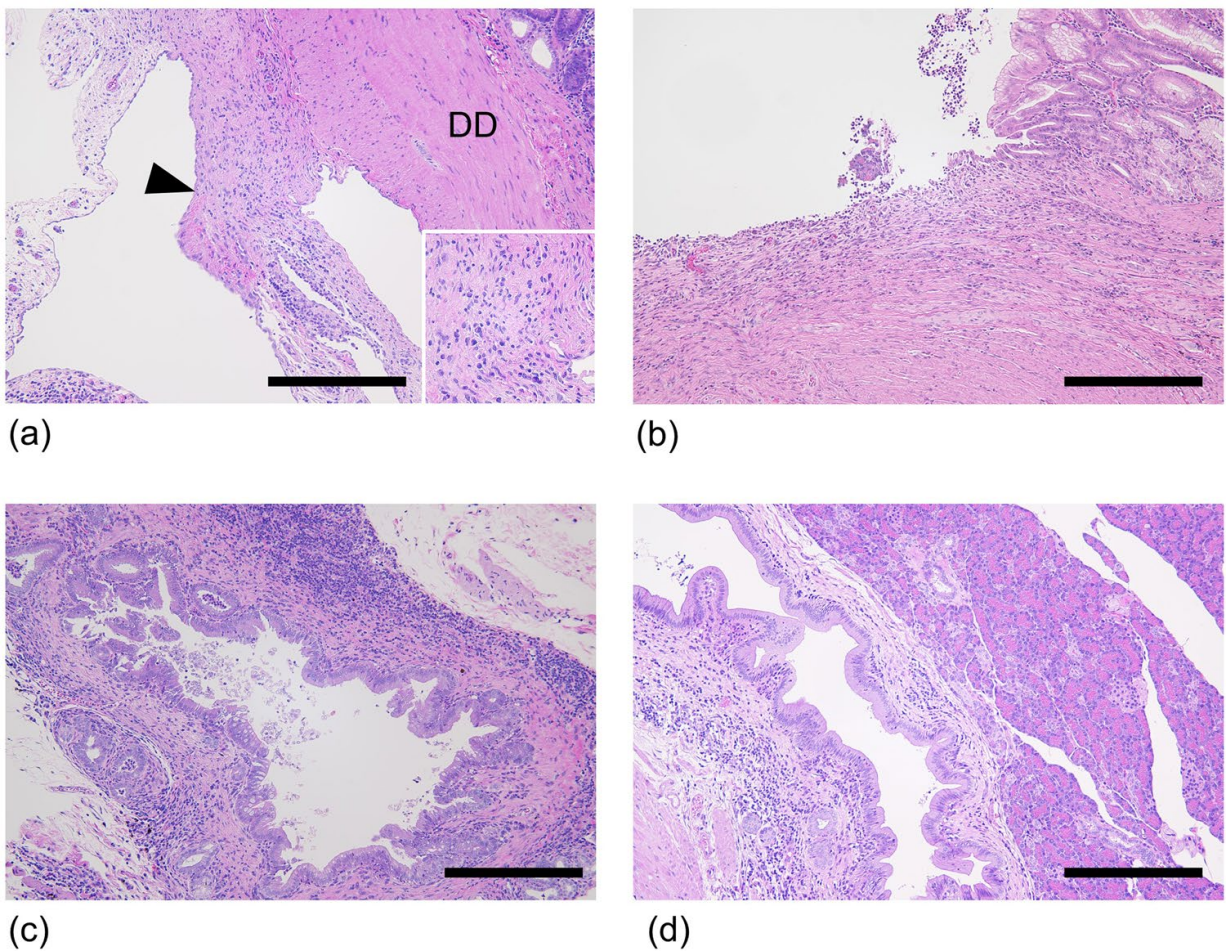
Histopathological appearance of cases with duodenal dilation. (**a**) Diseased case (case no. 8). Chronic peritonitis with proliferation of connective tissue between the duodenum and the colon was seen (arrowhead). Infiltration of lymphocytes and plasma cells was seen in the connective tissue. *Inset*: High-power magnification view of connective tissue. [DD: descending duodenum; Bar = 300 µm] (**b**) Diseased case (case no. 13). An ulcer was seen at the inferior flexure. Inflammatory cells infiltration (mainly lymphocytes, plasma cells, and eosinophils) and severe fibrosis were seen. Bar = 300 µm. (**c**) Diseased case (case no. 11). Cholecystitis with infiltration of inflammatory cells (mainly lymphocytes and plasma cells) was observed. Bar = 300 µm. (**d**) Diseased case (case no. 8). Pancreatic ductitis with infiltration of lymphocytes and plasma cells was seen. Bar = 300 µm.

Histopathological examination revealed chronic peritonitis with proliferation of connective tissue between the duodenum and the colon in all 8 animals evaluated (Fig. 3a). In 7 of 8 animals, ulcers were seen in the inferior flexure. Inflammatory infiltration (mainly lymphocytes, plasma cells, and eosinophils) and mild to severe fibrosis were also seen (Fig. 3b). In addition, histopathological analysis revealed cholangitis/cholecystitis (6 of 8) (Fig. 3c), chronic lymphocytic enteritis (4 of 8), pancreatic ductitis (2 of 8) (Fig. 3d), and liver abscess (1 of 8) (Table 1).

### Contrast radiography

**Figure 4:**
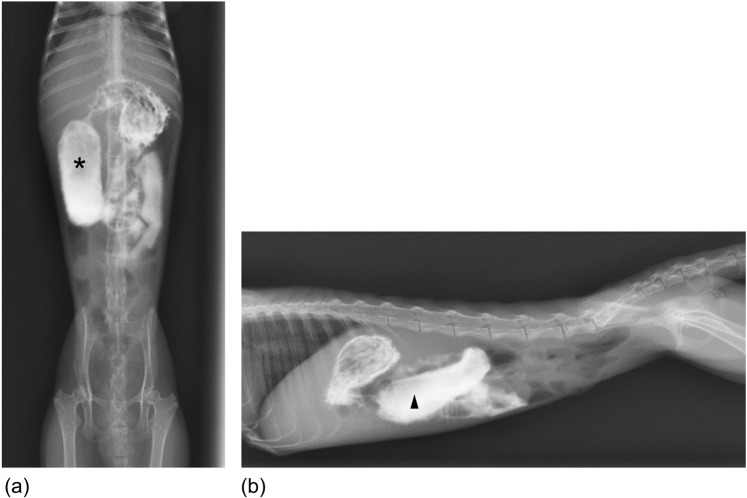
Contrast radiography of a diseased case (case no. 12). (**a**) Dilated duodenum was seen on the ventrodorsal (VD) view (asterisk). (**b**) Dilated duodenum was seen on the right-to-left lateral recumbent (RL) view (arrowhead).

Contrast radiography revealed dilated duodenums in the right side of the abdomen on left-to-right lateral recumbent (LR) and ventrodorsal (VD) views (Fig. 4). Contrast radiology could differentiate between diseased and control marmosets, with 100% sensitivity, specificity, positive predictive value, and negative predictive value (postmortem findings were used for reference) (Tables 2 and 3). Time taken for contrast radiography was ~60 min per animal.

**Table 2:** Accuracy of imaging tests in identifying duodenal dilation in marmosets (duodenal dilation cases vs control animals).

Imaging test	Sensitivity	Specificity	PPV^a^	NPV^b^	P value
Contrast radiography (n = 11)	100% (5/5)	100% (6/6)	100% (5/5)	100% (6/6)	<0.001
Plain radiography (n = 36)	50% (7/14)	100% (22/22)	100% (7/7)	75.9% (22/29)	<0.001
Ultrasonography (n = 34)	91.7% (11/12)	90.9% (20/22)	84.6% (11/13)	95.2% (20/21)	<0.001
Combination of plain radiography and ultrasonography (n = 34)	100% (12/12)	90.9% (20/22)	85.7% (12/14)	100% (20/20)	<0.001

^a^PPV = positive predictive value, ^b^NPV = negative predictive value.

**Table 3:** Results of imaging tests for marmosets with duodenal dilation.

Case No.	Contrast radiography^a^	Plain radiography^a^	Ultrasonography^a^	Combination of plain radiography and ultrasonography^a^
1	NA	Detect	NA	NA
2	NA	Not detect	Detect	Detect
3	NA	Detect	NA	NA
4	NA	Detect	Detect	Detect
5	NA	Detect	Not detect	Detect
6	NA	Detect	Detect	Detect
7	Detect	Detect	Detect	Detect
8	NA	Not detect	Detect	Detect
9	Detect	Not detect	Detect	Detect
10	NA	Not detect	Detect	Detect
11	Detect	Not detect	Detect	Detect
12	Detect	Not detect	Detect	Detect
13	NA	Detect	Detect	Detect
14	Detect	Not detect	Detect	Detect

^a^Detect = Duodenal dilation was detected; Not detect = Duodenal dilation was not detected.

### Screening tests (plain radiography and ultrasonography)

**Figure 5:**
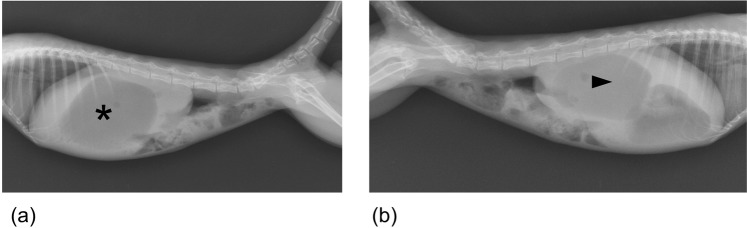
Plain radiography of a diseased case (case no. 13). (**a**) The duodenum was not detected by plain radiography on the left-to-right lateral recumbent (LR) view. Gas was located in the gastric corpus (asterisk). (**b**) The duodenum was detected by plain radiography on the right-to-left lateral recumbent (RL) view. Gas was located in the duodenum (arrowhead).

In control animals, duodenums could not be identified using plain radiography. Similarly, it was difficult to detect the duodenum using plain radiography in most of the diseased animals. In some cases, dilated duodenums were detectable because they were filled with gas. The right-to-left lateral recumbent (RL) views helped to identify the syndrome as they revealed gas in the displaced pylorus and enlarged duodenum (Fig. 5). Time taken for plain radiography was ~5 min per animal.

**Figure 6:**
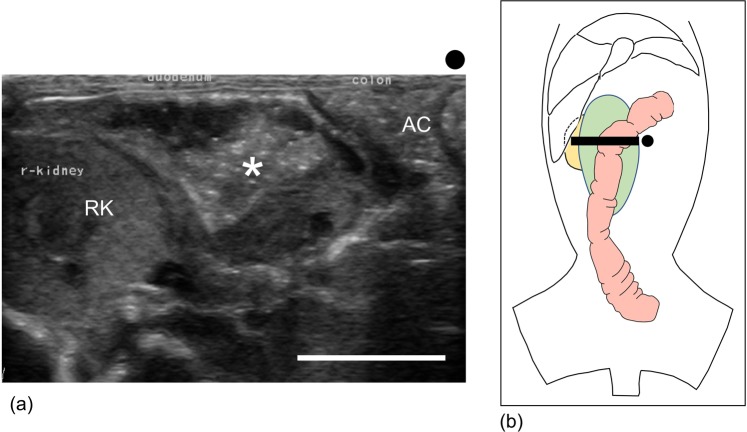
Ultrasonography of a diseased case (case no. 8). (**a**) Ultrasonography in transverse section images of the descending duodenum (asterisk), ascending colon (AC), and right kidney (RK). Ultrasonography revealed a dilated duodenum on the right side of the abdomen. The dilated duodenum contained abundant undigested food. The black mark indicates the transducer orientation. Bar = 10 mm. (**b**) The ventrodorsal anatomical schema showing the probe location (black line). The black mark indicates the transducer orientation. The anatomical schema was drawn by Ms. Chia-Ying Lee. [green organ: descending duodenum; red organ: ascending colon, and transverse colon; yellow organ: right kidney.

Ultrasonography was suitable for diagnosing duodenal dilation, except in marmosets with large amounts of bowel gas. In normal marmosets, the 5 sonographic layers of duodenum could be seen clearly, i.e. hyperechoic lumen/mucosa interface, hypoechoic mucosa, hyperechoic submucosa, hypoechoic muscularis propria, and hyperechoic serosa. The normal duodenums were mostly empty and constricted as previously reported^15^. The maximum duodenal diameters were approximately 4–6 mm in normal animals. In 11 out of 12 diseased cases, ultrasonography revealed dilated duodenums containing abundant fluid or content mixture (Fig. 6). Gas inside the intestines was a critical artefact for diagnosis; we wrongly diagnosed 2 control animals with abundant GI gas. Time taken for ultrasonography was 5–10 min per animal. When used in tandem, plain radiography and ultrasonography identified 100% of the marmosets with duodenal dilation (Tables 2 and 3).

## Discussion

In this study, we examined the clinical and pathological findings of a novel GI disease in marmosets characterised by duodenal dilation. Based on these results, we propose the designation ‘marmoset duodenal dilation syndrome’ for this disease, with the criterion for duodenal dilatation being a maximum diameter of the descending duodenum > 12 mm. Further, this disease indicates the following features: (1) dilation of the descending duodenum and filling with a mixture of gas and fluid; (2) adult incidence (ranging from 26 to 128 months); (3) no sex predilection; (4) clinical signs included vomiting, weight loss, and bloating; and (5) poor prognosis. The syndrome was diagnosed in 21.9% of necropsy cases and was one of the main life-threatening diseases in the CIEA marmoset colony. The abovementioned clinical signs in addition to hypoalbuminaemia and hypochloraemia, may reflect duodenal obstruction. We suspect aspiration pneumonitis, dehydration, electrolyte imbalance, and metabolic alkalosis as the major causes of death in this syndrome. The duodenal obstruction is irreversible, and all diseased marmosets showed a poor prognosis.

To our knowledge, duodenal dilation is very rare in humans or animals other than marmosets. In humans, superior mesenteric artery syndrome is a rare disease characterised by duodenal obstruction and dilation due to compression of the superior mesenteric artery^16^. By contrast, diseased marmosets did not show any superior mesenteric artery compression. In nonhuman primates, acute gastric dilation is an acute and severe condition^13,14^, while all 14 diseased cases in this study showed characteristically chronic duodenal dilation. However, 5 of the diseased cases also exhibited gastric dilation in addition to the duodenal dilation. This syndrome has devastating effects on marmoset health and can potentially hinder long-term research projects if diagnosed late in the clinical course of the disease. Therefore, the syndrome should be diagnosed as early as possible, before marmosets present with vomiting, weight loss, or bloating. We recommend that further studies confirm the occurrence and incidence of this disease in other marmoset facilities, and establish effective treatment plans for affected animals.

The cause of marmoset duodenal dilation syndrome is unknown, but it is most likely multifactorial. We consider that one pathogenesis of necessary condition of this syndrome was adhesive duodenal obstruction, where adhesions between the duodenum and colon hinder duodenal mobility. Though some control cases had adhesions (Fig. 2b, Adhesion score 1), all diseased cases showed severer adhesions, which were partial to direct adhesions (Fig. 2c,d, Adhesion score 1–2). Moreover, detachment of the adhesion released the duodenal obstruction in 6 necropsy cases. These findings suggest that the syndrome is caused by adherent obstruction secondary to chronic peritonitis. We could not identify the cause of the chronic peritonitis; however, cholangitis, cholecystitis, chronic lymphocytic enteritis, and pancreatic ductitis were suspected as possible primary lesions. In our institution, we observed pyogenic cholangitis due to *Escherichia coli* or *Pseudomonas aeruginosa* (data not shown); therefore, we should consider the involvement of bacterial infection in this syndrome. Another possibility of the pathogenicity of the duodenal obstruction is ulceration and fibrosis, causing stenosis at the inferior flexure and consequently, duodenal obstruction. Whether the duodenal ulcers observed in animals affected by the disease (Fig. 3b) were primary or secondary lesions was unclear. Future studies should aim to evaluate histopathological findings to identify primary and secondary lesions.

We also suspected that captive conditions were involved in enhancement of duodenal dilation, because occurrence of duodenal dilation was not reported in other marmoset facilities. Some reports showed that feeding conditions, such as overeating and consuming fermentable food, may cause worse acute gastric dilation in nonhuman primates^13,17^. Therefore, we should consider the involvement of feeding regimen in preventing duodenal dilation.

Contrast GI radiography is a valuable diagnostic test for evaluating mechanical ileus^18^. In our study, the duodenal dilations could be definitively diagnosed using contrast radiography. However, contrast GI radiography is time-consuming (~60 min per animal) and may further stress already diseased animals. Moreover, positive oral contrast agents (iodinated contrast medium or barium sulphfate suspensions) frequently cause side effects like nausea, vomiting, and diarrhoea^18^. Therefore, routine contrast GI radiography is not practical.

Single plain radiography has limited utility due to its low sensitivity in diagnosing the syndrome. It is widely available but has poor sensitivity to diagnosis intestinal obstruction^19^. To diagnose this syndrome using single plain radiography, gas inside the gastro-duodenum is necessary. Compared to radiography, ultrasonography is a useful, non-invasive diagnostic system for screening tests^15,19^. The sensitivity of ultrasonography was higher than that of plain radiography in this study, but it was difficult to detect the duodenum using ultrasonography in cases with abundant GI gas. The training of ultrasound technologists is crucial, because the precision of the ultrasound diagnosis depends on the experience and skill of the technologists. Furthermore, it is important to correlate ultrasound results with other diagnostic modalities to improve its diagnostic accuracy.

In this study, we established that a combination of plain radiography and ultrasonography without aneasthesia, can be used as a screening method for diagnosing duodenal dilation. The use of two imaging modalities is recommended for screening to improve the detection rate. If the screening tests are inconclusive, contrast radiography should be performed to confirm the diagnosis. Generally, for radiologic and ultrasonographic examination, anesthesia is recommended in order to minimize stress and the risk of injury, as well as to optimise the examination^15^. However, we do not recommend anaesthesia for animals with duodenal dilation, because it can cause acute vomiting and possibly aspiration. Future studies should investigate safe anesthetic techniques that can minimise the stress for animals with duodenal dilation.

In conclusion, we propose a novel GI disease ‘marmoset duodenal dilation syndrome’ characterised by an unexplained obstruction at the inferior flexure of the duodenum, ultimately resulting in duodenal dilation. We suspect the involvement of peritonitis, cholangitis, cholecystitis, chronic lymphocytic enteritis, and pancreatic ductitis in the pathogenesis of the syndrome. We established that imaging techniques such as contrast GI radiography, plain radiography, and ultrasonography without anesthesia can be used to diagnose duodenal dilation. Additional pathological analyses for primary lesions may provide valuable information regarding the pathogenic mechanisms of this syndrome.

## Methods

### Animals

This study was performed in strict accordance with the Regulations for Animal Experimentation of CIEA, which are based on the Guidelines for Proper Conduct of Animal Experiments (Science Council of Japan, 2006). The animal experiment protocol was approved by the Institutional Animal Care and Use Committee of the Central Institute for Experimental Animals (approval no. 16002 A).

Marmosets at CIEA were housed in family groups, in pairs or singly depending on experimental and veterinary care requirements. The cages measured 409–820 × 610 × 728–1578 mm and included wooden perches and a wooden resting place for environmental enrichment. They were positioned facing each other to allow the animals to communicate visually and vocally. The animal rooms were conditioned at 26–28 °C and 40–60% humidity with a 12 h:12 h light/dark cycle. The animals were fed a commercial New World primate diet (CMS-1M; CLEA Japan Inc., Tokyo, Japan) with added ascorbic acid (Nacalai Tesque Inc., Kyoto, Japan), vitamins A, D3, and E (Duphasol AE3D; Kyoritsu Seiyaku Co Ltd, Tokyo, Japan), and honey (Nihonhatimitsu, Gifu, Japan) and tap water ad libitum. In addition to the normal diets, gum arabic, sponge cakes, biscuits, marshmallows, or apple jelly were fed to the animals. Animals were checked multiple times daily by experienced animal keepers and/or veterinarians and were weighed every month. The animals were negative for *Salmonella* spp., *Shigella* spp. and *Yersinia pseudotuberculosis* in yearly monitoring.

Retrospective analyses for duodenal dilation in marmosets were conducted by searching the CIEA necropsy records for the years 2016–2018. Excluding neonates and research necropsies, 224 common marmosets were necropsied by veterinarians during this period. These animals included 108 males and 116 females, and their ages ranged from 6 to 204 months (average: 93 months). Of the 224 necropsied marmosets, 132 animals were euthanised and the other 92 animals died naturally.

Table 1 presents the clinical and pathological findings of the 14 marmosets with duodenal dilation that were selected for evaluation. One marmoset (case no. 5) that showed acute collapse during preparation for euthanasia was recorded as a natural death. The remaining 13 animals were deeply anaesthetised with 50 mg/kg ketamine, 4 mg/kg xylazine, and 1–3% isoflurane and then euthanised by exsanguination from the abdominal aorta due to their moribund condition (Table 1). Following both the natural death and the euthanasia, all animals immediately underwent a thorough necropsy.

As a control group, 22 necropsied animals without duodenal dilation (9 males and 13 females) aged 25–192 months (average: 100 months) were included (Table 1). They included MWS (n = 8), *Clostridium difficile* infection (n = 2), tumour (n = 2), and other (n = 10). Control animals were not included in every comparison.

### Adhesion scoring

Adhesion scoring between the descending duodenum and ascending colon was estimated in the necropsies. The semi-quantitative adhesion scoring was defined as follows:

Adhesion score 0 - no adhesions between the duodenum and the colon

Adhesion score 1 - partial adhesions

Adhesion score 2 - tight and direct adhesions

### Blood tests

Blood samples obtained from the euthanised marmosets (case nos. 1, 3, 4, 6–14) were used. Complete blood count was analysed using a Sysmex XT-2000i (Sysmex Corporation, Kobe, Japan). The clinical chemistry was analysed using DRI-CHEM 7000 (Fujifilm Corporation, Tokyo, Japan).

### Abdominal radiography (plain and contrast)

Abdominal radiography was performed without anesthesia, using a digital radiography system (Carestream Vita CR system; Carestream Health, NY, USA) with 55 kV and 1.2 mAs for marmosets.

Plain abdominal radiography was performed in the LR, RL, and VD positions.

Contrast GI radiography was performed as the reliable diagnostic test in the LR and VD positions for 5 diseased marmosets (case nos. 7, 9, 11, 12, 14). Gastrografin (Bayer AG, Leverkusen, Germany) was intragastrically administered to each marmoset at a volume of 1.5 mL/head.

### Abdominal ultrasonography

Transabdominal ultrasound examinations were performed without anesthesia using a ProSound Alpha 7 ultrasound system (Hitachi Aloka Medical, Tokyo, Japan) with an 8 MHz linear probe. The maximum diameters of the descending duodenum were estimated using the transverse images.

### Histopathological analysis

Histopathological examinations were performed on samples from 8 marmosets (case nos. 2, 3, 8, 10–14). All major organs were fixed in 10% neutral-buffered formalin, embedded in paraffin, cut, and stained with hematoxylin and eosin.

### Statistical analyses

Comparison of adhesion scoring between animals with and without duodenal dilation was performed using the unpaired T test. We compared the imaging tests and gross pathological findings of necropsy, and calculated the sensitivity, specificity, positive predictive value, and negative predictive value of the imaging tests; this was done using two-tailed Fisher’s exact tests. P values of <0.05 were considered to be statistically significant. All statistical analyses were performed on GraphPad Prism 7 (GraphPad Software Inc., CA, USA).

## Supplementary information

Supplementary information

## Data Availability

All data generated during and/or analysed during the current study are available from the corresponding author on reasonable request.

## References

[1] Sasaki E (2009). Generation of transgenic non-human primates with germline transmission. Nat..

[2] Sato K (2016). Generation of a nonhuman primate model of severe combined immunodeficiency using highly efficient genome editing. Cell Stem Cell.

[3] Marini, R., Wachtman, L., Tardif, S., Mansfield, K. & Fox, J. (eds.) *The Common Marmoset in Captivity and Biomedical Research*, 1st edn (Elsevier Science Publishing Co Inc, 2018).

[4] Takahashi T (2014). Birth of healthy offspring following ICSI in *in vitro*-matured common marmoset (Callithrix jacchus) oocytes. PLoS One.

[5] Ludlage E, Mansfield K (2003). Clinical care and diseases of the common marmoset (Callithrix jacchus). Comp. Med..

[6] Yokouchi Y, Imaoka M, Sayama A, Jindo T, Sanbuissho A (2013). Inflammatory fibroid polyp in the duodenum of a common marmoset (Callithrix jacchus). Toxicol. Pathol..

[7] Miller AD (2010). Small intestinal adenocarcinoma in common marmosets (Callithrix jacchus). Vet. Pathol..

[8] Logan AC, Khan KN (1996). Clinical pathologic changes in two marmosets with wasting syndrome. Toxicol. Pathol..

[9] Baxter VK (2013). Serum albumin and body weight as biomarkers for the antemortem identification of bone and gastrointestinal disease in the common marmoset. PLoS One.

[10] Kuehnel F (2013). The influence of gluten on clinical and immunological status of common marmosets (Callithrix jacchus). J. Med. Primatol..

[11] Otovic P, Smith S, Hutchinson E (2015). The use of glucocorticoids in marmoset wasting syndrome. J. Med. Primatol..

[12] Sousa MB, Leao AC, Coutinho JF, de Oliveira Ramos AM (2008). Histopathology findings in common marmosets (Callithrix jacchus Linnaeus, 1758) with chronic weight loss associated with bile tract obstruction by infestation with Platynosomum (Loos, 1907). Primates.

[13] Stein FJ, Lewis DH, Stott GG, Sis RF (1981). Acute gastric dilatation in common marmosets (Callithrix jacchus). Lab. Anim. Sci..

[14] Abee, C. R., Mansfield, K., Tardif, S. & Morris, T. (eds.) *Nonhuman Primates in Biomedical Research*, 2nd edn, Vol. 2 (Academic Press, 2012).

[15] Wagner WM, Kirberger RM (2005). Transcutaneous ultrasonography of the abdomen in the normal common marmoset (Callithrix jacchus). Veterinary Radiology Ultrasound.

[16] Unal B (2005). Superior mesenteric artery syndrome: CT and ultrasonography findings. Diagn. Interv. Radiol..

[17] Pond CL, Newcomer CE, Anver MR (1982). Acute gastric dilatation in nonhuman primates: review and case studies. Veterinary Pathol..

[18] J. K Kealy, H MA & John P. Graham Diagnostic Radiology and Ultrasonography of the Dog and Cat. (Saunders, 2011).

[19] Horton KM, Fishman EK, Gayler B (2008). The use of iohexol as oral contrast for computed tomography of the abdomen and pelvis. J. Comput. Assist. Tomogr..

